# Low back pain and psychological distress according to the job tenure among electricians

**DOI:** 10.1192/j.eurpsy.2024.1710

**Published:** 2024-08-27

**Authors:** I. Sellami, A. Feki, A. Abbes, M. Hajjaji, S. Baklouti, M. L. Masmoudi, K. Jmal Hammami

**Affiliations:** ^1^Occupational medicine, Hedi Chaker Hospital; ^2^ Medecine univeristy; ^3^Rheumatoloy, Hedi Chaker Hospital, Sfax, Tunisia

## Abstract

**Introduction:**

Low back pain (LBP) is common among electricians caused by work conditions. Even when symptoms are short-term and not medically serious, LBP can be associated with psychological distress.

**Objectives:**

This study aimed to assess the link between LBP and psychological distress according to the job tenure among electricians.

**Methods:**

The study was conducted in a group from a Tunisian Electricity society. Data were gathered between January-June 2022 using a self-administered questionnaire including socio-professional characteristics, the Nordic musculoskeletal questionnaire during the last 12 months and Kessler Psychological Distress Scale (K6). Our population was divided into two groups according to job tenure. The first group (G1) consisted of electricians with less than ten years of job tenure and the second (G2) consisted of electricians with more than ten years of seniority.

**Results:**

G1 consisted of 10 participants with a mean age of 30.6 ± 6.7 years and with average job tenure of 3.3 ± 1.1 years. G2 consisted of 64 participants with a mean age of 40.7 ± 10.3 years and average job tenure of 17.4 ± 10.9 years. According to the Nordic musculoskeletal questionnaire, LBP during the last 12 months was present in the first and the second group in 30.8% and 14.3% of participants, respectively.

The proportion of respondents with high levels of psychological distress (K6 score of 13 or greater) in the first and the second groups was 10 % and 9.4% of participants, respectively. The presence of low back pain during the last 12 months was significantly associated with a high score of K6 in the second group (p < 0.05).

**Conclusions:**

From the results of this study, we conclude that LBP was associated with psychological distress when the job tenure is high. Therefore, the prevention of LBP should go through programmes to build ergonomically safe working conditions to enhance the mental health of electricians.

**Disclosure of Interest:**

None Declared

